# Field Evidence of Endogenous Vitamin D Synthesis in Atlantic Salmon Induced by Natural Sunlight

**DOI:** 10.1155/anu/3823472

**Published:** 2025-12-20

**Authors:** Christina Aspaas Husebø, Kjetil Berge, Frederike Keitel-Gröner, Eirik Hoel, Johan Rennemo, Margunn Sandstad, Kristine Marie Bjerkestrand, Lukas Lorentzen, Eirik Welde, Thea Morken, Ingunn Stubhaug, Julia Mullins, Håvard Bjørgen, Thea Bossum, Cato Brede, David Lausten Knudsen

**Affiliations:** ^1^ Skretting AS, Sjøhagen 6, N-4016, Stavanger, Norway; ^2^ Nordlaks AS, Industriveien 14, N-8450, Stokmarknes, Norway; ^3^ Skretting Aquaculture Innovation, Sjøhagen 6, N-4016, Stavanger, Norway; ^4^ Unit of Anatomy, Faculty of Veterinary Medicine, Norwegian University of Life Sciences, N-1433, Ås, Norway, nmbu.no; ^5^ Department of Chemistry, Bioscience and Environmental Engineering, University of Stavanger, N-4036, Stavanger, Norway, uis.no; ^6^ FishLab AS, Storhaug Allé 8, N-4015, Stavanger, Norway; ^7^ Department of Medical Biochemistry, Stavanger University Hospital, N-4068, Stavanger, Norway, helse-stavanger.no

**Keywords:** Atlantic salmon, cholecalciferol, endogenous synthesis, fish, sunlight, UV light, vitamin D

## Abstract

Fish are believed not to synthesize vitamin D through UV exposure but to meet their requirements from dietary sources. The high vitamin D levels found in many fish species are thought to originate from UV‐induced synthesis in plankton, with vitamin D subsequently accumulating through the aquatic food chain. Atlantic salmon is a rich dietary source of vitamin D, but limited data exist on circulating vitamin D levels. To address this, plasma levels of the three main vitamin D metabolites were measured in Atlantic salmon from Norwegian fish farms using mass spectrometry. Here, we show that salmon reared in open sea pens have significantly higher vitamin D levels than those raised indoors. Monitoring an outdoor farm over 18 months revealed a distinct seasonal pattern between vitamin D levels and day length. In a follow‐up experiment, indoor‐raised fish were divided into two groups: one remained indoors, while the other was transferred outdoors for 52 days. Both groups received the same commercial feed, yet the outdoor group exhibited a fivefold increase in whole‐body vitamin D content. These surprising findings provide field evidence of endogenous vitamin D synthesis in fish induced by natural sunlight. This discovery could have important implications for aquaculture, emphasizing the risk of suboptimal vitamin D levels in farmed fish when sunlight exposure is limited.

## 1. Introduction

The first evidence of vitamin D was discovered by Sir Edward Mellanby in 1919, who identified it as a nutritional substance that could cure rickets. In 1922, Elmer McCollum further investigated this compound and named it vitamin D. Later, Adolf Windaus revealed its molecular structure, a breakthrough that earned him the Nobel Prize in Chemistry in 1928 [1–3]. Vitamin D was found to be a secosteroid, and it has been shown to exist in more than 30 different forms [4, 5].

The inactive precursor cholecalciferol (D3) is produced by most terrestrial animals when 7‐dehydrocholesterol in the skin is exposed to UVB light from the sun. Additionally, D3 can be absorbed through the intestine from foods and supplements. D3 is converted in the body to 25‐hydroxycholecalciferol (25(OH)D3) and finally to its biologically active form, 1*α*,25‐dihydroxycholecalciferol (1,25(OH)_2_D3), which acts as an endocrine hormone [6, 7]. Ergocalciferol (D2) is a vitamin D form that is produced by certain plants and microorganisms. This form of vitamin D can also contribute to vitamin D activity, but the circulating levels of D2 metabolites in fish are normally significantly lower than metabolites from D3 and will be out of scope for this study [8].

The most abundant vitamin D metabolite in human plasma is 25(OH)D3, with a normative level of 30–100 ng per mL (75–250 nmol per liter). The circulating level of D3 is typically 4–40 ng per mL (10–100 nmol per liter), whereas 1,25(OH)_2_D3 is present only in the picogram‐per‐milliliter range (<0.2 nmol per liter). The half‐lives of these three metabolites are in the order of weeks, days, and hours, respectively, making 25(OH)D3 both the most abundant and most stable vitamin D metabolite in human plasma [9–15].

Although the active metabolite 1,25(OH)_2_D3 might be expected to be an ideal biomarker for vitamin D status, it is not. This is not primarily due to its low abundance and rapid clearance from circulation, but rather because of its intricate regulation. As a patient becomes vitamin D deficient, intestinal calcium absorption decreases, and lower levels of free calcium in the blood signal increased secretion of parathyroid hormone, which in turn leads to increased activation of vitamin D. In fact, elevated levels of 1,25(OH)_2_D3 are often seen in individuals suffering from vitamin D deficiency [13].

Circulating 25(OH)D3, on the other hand, has been shown to correlate strongly with classic signs of vitamin D deficiency such as impaired bone mineralization, and 25(OH)D3 is today considered the best biomarker for vitamin D status in humans [13, 16]. Due to its high abundance, long half‐life, and the required hydroxylation step from D3, immediate postprandial effects on 25(OH)D3 levels from dietary D3 absorption are relatively minor [17]. As a result, fasting is not crucial before measuring 25(OH)D3, which simplifies clinical application. However, regular dietary supplementation of D3 can, over time, improve the level of circulating 25(OH)D3 significantly in vitamin D‐deficient individuals [18, 19].

The primary physiological role of vitamin D is to control calcium and phosphate homeostasis by regulating intestinal uptake, mobilization from bone, and reabsorption of calcium and phosphate in the kidneys [5, 20]. The mechanism of action of the vitamin D hormone is mediated by the vitamin D receptor (VDR) protein, which was discovered and characterized by Haussler’s group [2]. The first evidence of novel activities of vitamin D, besides controlling mineralization and skeletal growth, emerged when VDR was unexpectedly found to be present in many cell types not involved in calcium and phosphate homeostasis, such as pancreatic and ovarian cells, keratinocytes in the skin, and immune cells like monocytes and lymphocytes [5, 21]. Indeed, recent clinical and epidemiologic studies indicate that vitamin D levels not only control mineral homeostasis but also play a vital role in the immune system. These studies further suggest a possible link between low vitamin D levels and an increased risk of several serious conditions such as muscle weakness, diabetes, hypertension, cardiovascular disease, autoimmune diseases, inflammatory conditions, and several types of cancer [4, 6, 14, 22].

The prevailing consensus is that fish, unlike most terrestrial vertebrates, do not synthesize vitamin D through UV exposure but instead obtain it from dietary sources [23]. It is hypothesized that the high vitamin D levels observed in many fish species originate from UV‐induced vitamin D synthesis in zooplankton and phytoplankton, which is then accumulated and transferred through the aquatic food chain [23, 24].

Atlantic salmon is considered to be one of the richest dietary sources of vitamin D, with a typical level of 6–9 µg D3 per 100 g of fillet in farmed salmon [25]. However, only limited data are available on the typical level of circulating vitamin D in plasma [26, 27]. This study was conducted to gather more data on circulating vitamin D metabolites in farmed Atlantic salmon and to explore whether these levels varied between farms or throughout the production cycle. The findings could guide adjustments to dietary vitamin D levels in feed if significant differences were found. Additionally, the levels of vitamin D metabolites in whole‐body samples of juvenile salmon were examined.

## 2. Methodology

### 2.1. Fish Samples

**Figure 1:**
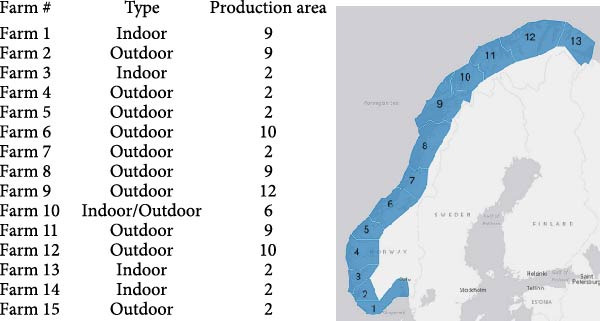
Overview of farms and geographical location along the Norwegian coast (the map of production areas was adapted from the Directorate of Fisheries in Norway). More information on fish sizes and dates of samplings can be found in the supporting information data file.

Plasma samples from Atlantic salmon (*Salmo salar*) were collected from 15 different fish farms in Norway. The geographical location of the farms (production areas) is shown in Figure 1.

Fish were euthanized by an overdose of anesthesia (Finquel vet., MSD Animal Health Norway AS, Norway) before sampling. Blood was sampled from the caudal vein using 4‐mL lithium‐heparinized vacuum tubes. Tubes were gently inverted five times to allow adequate mixing with the coated anticoagulant. Plasma was separated on‐site by centrifugation at 2500 × *g* for 5 min. Samples were transferred to 2 mL polypropylene microcentrifuge tubes and kept at 40°C prior to analysis. From a hatchery (Farm 10), where fish were reared both indoors and outdoors, whole juvenile fish (5 individual fish per group) were sampled to measure the content of stored vitamin D metabolites in the whole body. Fish were euthanized as described previously, homogenized, and kept at −40°C prior to analysis.

### 2.2. Chemicals and Solutions

Analytes and isotopically labeled internal standards were from Merck (USA): 99.7% D3, 99.4% 25(OH)D3, 98% D2 (6,19,19‐d3), and 99.7% 25(OH)D3 (26,26,26,27,27,27‐d6). Analytes and isotopically labeled internal standards were from Toronto Research Chemicals (Canada): 95% 1,25(OH)2D3 and 99.4% 1,25(OH)_2_D3 (26,26,26, 27,27,27‐d6). LC‐MS grade methanol and acetonitrile (ACN), ethanol, anhydrous ACN, and ammonium sulfate were from VWR International (Radnor, PA, USA). Water was type I, purified to 18.2 MOhm. Derivatization reagent 4‐phenyl‐1,2,4‐triazoline‐3,5‐dione (PTAD) was from Tokio Chemical Industry (Japan). A saturated solution of ammonium sulfate (~4 mol per liter (NH_4_)_2_SO_4_) was made by adding more (NH_4_)_2_SO_4_ than could be dissolved in water at room temperature. The internal standard working solution was prepared in ACN with 200 nmol/L D2 (6,19,19‐d3), 451 nmol per liter 25(OH)D3 (26,26,26,27,27,27‐d6), and 237 nmol per liter 1,25(OH)_2_D3 (26,26,26, 27,27,27‐d6).

### 2.3. Sample Preparation

**Figure 2:**
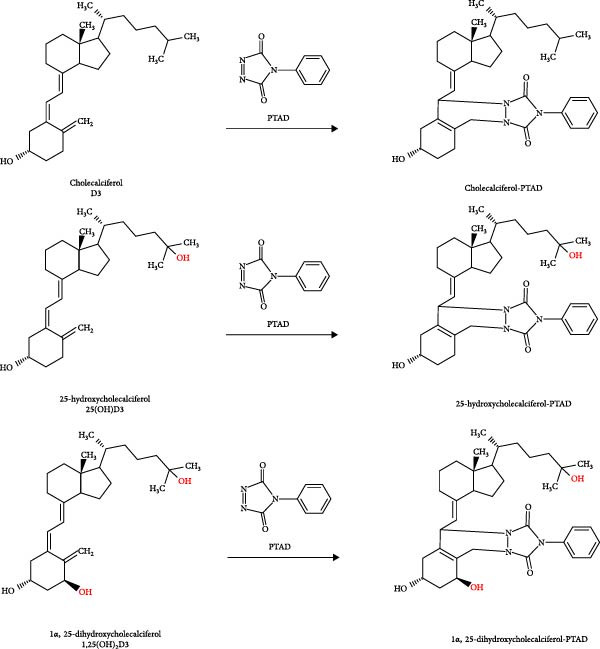
Molecular structures of the three main vitamin D metabolites analyzed in the present study. PTAD was used as a derivatization reagent to increase ionization efficiency, thereby enhancing the sensitivity of the LC‐MS/MS method.

Plasma samples (100 µL) were mixed with the internal standard working solution (100 µL) and subjected to salting‐out assisted liquid–liquid extraction (SALLE). First, the samples were diluted with 300 µL ACN and mixed with 100 µL of saturated ammonium sulfate to induce phase separation. Next, the vials were centrifuged at 2000 × g for 5 min to achieve clear separation of the solvent phase. Finally, a derivatization step was performed by adding 20 µL of PTAD dissolved in anhydrous ACN (20 mg per mL) to the solvent phase and incubating the solution for 15 min at room temperature (Figure 2).

Samples of whole‐body homogenate (0,1–0,2 g) were mixed with internal standard working solution (100 µL) and then hydrolyzed by addition of ethanol (100 µL) and 4M KOH (200 µL) followed by incubation for 60 min at 60°C. After cooling to room temperature, liquid–liquid extraction was done by adding heptane (700 µL), shaking, and centrifugation at 2000 × *g* for 5 min. The solvent (500 µL) was removed and evaporated under nitrogen gas at 35°C before a final derivatization step was done by resuspending samples in 300 µL PTAD dissolved in anhydrous ACN (0.5 mg per mL). Samples were incubated for a minimum of 15 min at room temperature before analyses.

### 2.4. LC‐MS/MS

Derivatized plasma sample extracts were analyzed by using an Acquity UPLC coupled with a Quattro Premier XE tandem mass spectrometer (Waters, Milford, MA, USA). 10 µL of sample extract was injected onto an Acquity BEH C18 reversed‐phase column with 2.1 mm ID, 100 mm length, and 1.7 µm particle size (Waters). The mobile phase consisted of (A) 0.2% (v/v) concentrated ammonium hydroxide in water mixed with (B) methanol at a flow rate of 0.20 mL/min. The linear step gradient was as follows: 0 min (40% B), 0.2 min (72% B), 1.2 min (74% B), 1.6 min (76% B), 3.2 min (78% B), 3.3 min (99% B), 6.5 min (99%), 6.6 min (40% B), and 9.0 min (40% B).

Derivatized extracts of whole‐body homogenates were analyzed as described above for plasma samples, but the linear step gradient was modified as follows: 0 min (50% B), 0.2 min (75% B), 2.2 min (80% B), 2.5 min (90% B), 5.0 min (90% B), 5.1 min (99% B), 9.5 min (99%), 9.6 min (50% B), and 12.0 min (50% B).

Positive electrospray ionization (ESI+) with 3 kV capillary voltage was applied for MS/MS detection using multiple reaction monitoring (MRM) with parameters shown in Table 1. For plasma samples, the retention times were as follows: 5.8 min for D3, 4.95 min for 25(OH)D3, and 4.2 min for 1,25(OH)_2_D3. For whole‐body homogenate samples, the retention times were as follows: 6.5 min for D3 and 4.0 min for 25(OH)D3. 1,25(OH)_2_D3 was not quantified in samples of whole‐body homogenate.

**Table 1:** MRM detection parameters.

Analytes and internal standards	Molecular ion formula ^*a*^	Molecular ion *m/z*	Fragment ion *m/z*	Cone voltage (V)	Collision energy (eV)
D3	[C_35_H_49_N_3_O_3_+H]^+^	560.3	298.1	26	16
D2 (6,19,19‐d_3_)	[C_36_D_3_H_46_N_3_O_3_+H]^+^	575.4	301.1	26	16
25(OH)D3	[C_35_H_49_N_3_O_4_H–H_2_O+H]^+^	558.3	298.1	32	17
25(OH)D3 (26,26,26,27,27,27‐d_6_)	[C_35_D_6_H_43_N_3_O_4_–H_2_O+H]^+^	564.3	298.1	32	17
1,25(OH)_2_D3	[C_35_H_49_N_3_O_5_–H_2_O+H]^+^	574.3	314.1	35	15
1,25(OH)_2_D3 (26,26,26,27,27,27‐d_6_)	[C_35_D_6_H_43_N_3_O_5_–H_2_O+H]^+^	580.3	314.1	35	15

*Note:* Values are shown for PTAD derivatization products of vitamin D metabolites and internal standards.

^a^of PTAD derivatization product.

### 2.5. Method Performance

Calibration standard solutions were prepared with newborn calf serum (Hyclone, Cytiva, USA). Internal standard calibration and four quality control (QC) samples were included in all series of analysis. The QC samples were one pooled sample of fish plasma (Atlantic salmon), two samples of calf serum spiked with two different concentration levels, and one human serum‐based commercial control sample (SERO AS, Norway).

For measurement in fish plasma, the method limits of detection (LOD) were 0.7 nmol per liter for 1,25(OH)_2_D3, 0.3 nmol per liter for 25(OH)D3, and 0.2 nmol per liter for D3. For measurement in whole fish, the method LODs were 0.036 µg per 100 g for 25(OH)D3 and 0.008 µg per 100 g for D3. Method reproducibility for fish plasma (*n* = 38) was with coefficients of variation (CV) of 12% for D3 and 25(OH)D3 and 25% for 1,25(OH)_2_D3 at concentration levels of 311, 18, and 3 nmol per liter, respectively. Reproducibility of measurements (*n* = 45) in calf serum spiked with low levels (5 nmol per liter) was measured with CVs in the range of 9%–22%. Calf serum spiked with higher levels (40 nmol per liter) was measured with CVs in the range of 10%–13%. The human serum control sample had an assigned concentration level for 25(OH)D3 set to 53 nmol/L (acceptable range 40–67 nmol per liter). We measured 62 nmol per liter of 25(OH)D3 in this sample with a CV of 9% (*n* = 50). Hence, we observed satisfactory method reproducibility for D3 and 25(OH)D3 and acceptable bias for 25(OH)D3 against the human control sample. Reproducibility observed for repeated measurement of the fish plasma (*n* = 7) with the full‐body homogenate method was with CVs of 12% and 14% for D3 and 25(OH)D3, respectively. Further assessment of accuracy was done by testing method recovery by measurement of fish plasma without spiking (*n* = 4) and with spiking with low (*n* = 4) and high (*n* = 4) concentration levels. This showed the following mean recoveries at low and high spiking levels: 77% and 84% for D3, 83% and 122% for 25(OH)D3, and 80% and 125% for 1,25(OH)_2_D3. Accuracy for the fish homogenate method was assessed by similar spiking of a homogenized fish fillet sample. The following mean recoveries at low and high spiking levels were then observed: 114% and 104% for D3 and 81% and 109% for 25(OH)D3.

### 2.6. Data Analysis

Statistical analyses were performed using GraphPad Prism (version 10.4.2, GraphPad Software, San Diego, CA, USA). Welch’s unequal variances *t*‐test was used to compare groups with unequal variances or sample sizes, as it better controls Type I error in these cases than the standard Student’s *t*‐test [28, 29].

When multiple groups were compared, mean values were first analyzed using a one‐way analysis of variance (ANOVA) to assess overall differences among groups. When significant effects were detected, Tukey’s multiple comparisons test was applied as a post hoc test to identify pairwise differences between groups. To indicate statistical differences, groups sharing the same letter in the figures are not significantly different from each other, while groups with different letters differ significantly based on Tukey’s multiple comparisons test (*p* < 0.05).

## 3. Results

### 3.1. Initial Screening of 12 Fish Farms

Twelve Norwegian fish farms, three indoor and nine outdoor farms, were selected for the first screening. Results are summarized in Table 2. The outdoor pens in the fjords had a depth of about 30–40 m, and fish could move freely between the bottom and the surface. When the data were sorted between indoor and outdoor farms, a clear pattern emerged.

**Table 2:** Level of vitamin D metabolites in plasma (nmol per liter) from Atlantic salmon sampled at 12 different fish farms.

Farm #	Farm type (salinity)	Date dd/mm (sample size)	D3	25(OH)D3	1,25(OH)_2_D3
Farm 1	Indoor (FW)	22/05 (89)	15.7 ± 10.7	2.2 ± 1.1	1.0 ± 0.7
Farm 2	Outdoor (SW)	15/03 (34)	95.5 ± 29.4	11.8 ± 2.8	1.0 ± 0.5
Farm 3	Indoor (FW)	30/05 (40)	10.7 ± 6.7	2.8 ± 0.8	1.4 ± 1.0
Farm 4	Outdoor (SW)	29/09 (20)	95.1 ± 62.8	18.1 ± 3.8	1.1 ± 0.9
Farm 5	Outdoor (SW)	16/05 (20)	129.3 ± 74.1	14.1 ± 4.3	1.8 ± 1.4
Farm 6	Outdoor (SW)	07/06 (8)	180.7 ± 67.2	9.9 ± 4.6	3.8 ± 2.9
Farm 7	Outdoor (SW)	29/06 (9)	76.7 ± 13.9	16.1 ± 2.2	1.9 ± 0.9
Farm 8	Outdoor (SW)	31/10 (4)	54.5 ± 4.8	9.9 ± 1.0	1.9 ± 1.5
Farm 9	Outdoor (SW)	07/02 (20)	68.7 ± 70.6	7.4 ± 2.5	1.4 ± 1.0
Farm 10	Indoor (FW)	27/05 (9)	5.5 ± 1.4	1.9 ± 0.8	2.6 ± 1.3
Farm 11	Outdoor (SW)	06/09 (29)	173.2 ± 103.1	15.2 ± 5.4	5.0 ± 2.1
Farm 12	Outdoor (SW)	23/07 (11)	132.1 ± 23.7	17.3 ± 2.7	2.0 ± 1.1
Average for indoor farms (FW)		**(3)**	**10.6 ± 5.1**	**2.3 ± 0.5**	**1.7 ± 0.8**
Average for outdoor farms (SW)		**(9)**	**111.8 ± 44.9**	**13.3 ± 3.7**	**2.2 ± 1.3**
Welch’s *t*‐test ^∗^			*p* = 0.0001	*p* < 0.0001	*p* = 0.46

*Note:* Mean value ± standard deviation. Bold values indicate statistically significant differences between indoor and outdoor farms, determined using Welch’s *t*‐test (unequal variance *t*‐test).

Abbreviations: FW, freshwater (0–4 ppt); SW, seawater (28–34 ppt).

^∗^Unequal variance *t*‐test.

Both D3 and 25(OH)D3 were significantly higher in plasma samples from fish kept in outdoor pens, numerically 10 and 6 times higher, respectively, compared to values found in fish from indoor facilities. The level of 1,25(OH)2D3 also showed considerable variation between sites, but no statistical difference was found between indoor and outdoor farms in the first screening.

### 3.2. Vitamin D Levels Before and After Transfer to Outdoor Sea Pens

Some of the fish groups sampled at the 12 farms likely belonged to different genetic strains. To rule out the possibility that the lower levels of D3 and 25(OH)D3 observed in fish reared indoors were influenced by genetic differences, three fish groups were sampled both before and after being moved from indoor hatcheries to outdoor grow‐out facilities in the fjords. The third group was monitored closely by doing 4 samplings before transfer and 3 samplings after transfer. Results from these analyses are shown in Table 3. The plasma level of both D3 and 25(OH)D3 had increased dramatically in all groups at the first sampling after transfer to open sea pens. In group 3, a 16‐fold and 6‐fold increase in D3 and 25(OH)D3, respectively, was observed within just 26 days.

**Table 3:** Level of vitamin D metabolites in plasma (nmol per liter) in three different fish groups sampled before and after transfer to outdoor pens.

Fish group	Farm # (Farm type)	Sampling time (sample size)	D3	25(OH)D3	1,25(OH)_2_D3
Group 1	13 (Indoor)	114 days before (20)	7.7 ± 6.9	4.0 ± 3.1	2.3 ± 2.3
	4 (Outdoor)	29 days after (20)	95.1 ± 62.8	18.1 ± 3.8	1.1 ± 0.9

Group 2	13 (Indoor)	51 days before (20)	16.5 ± 9.3	5.0 ± 2.4	1.8 ± 1.2
	5 (Outdoor)	52 days after (20)	129.3 ± 74.1	14.1 ± 4.3	1.8 ± 1.4

Group 3	1 (Indoor)	54 days before (89)	15.7 ± 10.7	2.2 ± 1.1	1.0 ± 0.7
	1 (Indoor)	38 days before (116)	19.4 ± 9.7	1.6 ± 0.9	3.4 ± 2.3
	1 (Indoor)	23 days before (52)	19.1 ± 13.1	2.5 ± 0.9	5.9 ± 2.5
	1 (Indoor)	11 days before (28)	13.9 ± 8.1	2.5 ± 0.8	4.6 ± 2.3
	11 (Outdoor)	26 days after (90)	222.7 ± 86.4	16.3 ± 6.3	2.6 ± 1.9
	11 (Outdoor)	53 days after (29)	173.2 ± 103.1	15.2 ± 5.4	5.0 ± 2.1
	11 (Outdoor)	83 days after (88)	78.1 ± 20.7	13.2 ± 3.0	3.2 ± 1.6

*Note:* Mean value ± standard deviation. Group 1 came from Farm 13 and was transferred to Farm 4. Group 2 came from Farm 13 and was transferred to Farm 5. Group 3 came from Farm 1 and was transferred to Farm 11.

### 3.3. Effect of Salinity

**Figure 3:**
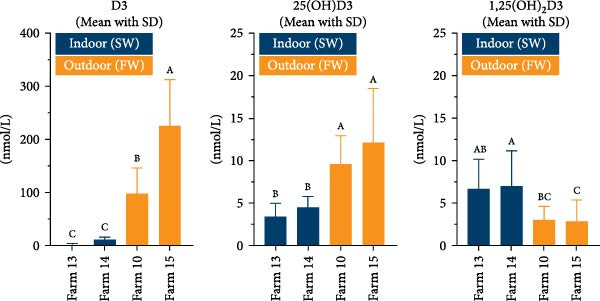
Vitamin D metabolites in plasma from fish reared indoors in seawater (SW) and outdoors in freshwater (FW), reported in nmol per liter. Number of fish analyzed: Farm 13 (*n* = 19), Farm 14 (*n* = 30), Farm 10 (*n* = 9), Farm 15 (*n* = 20). Data are presented as mean with standard deviation shown as upper error bars. Mean values per farm were compared using one‐way ANOVA and Tukey’s multiple comparisons test. Sites with different letters indicate statistically significant differences in mean values (*p* < 0.05).

It has previously been shown that the concentration of several blood parameters can differ between Atlantic salmon reared in freshwater and those reared in seawater [30]. To rule out the possibility that the observed differences in plasma vitamin D concentrations after transfer to outdoor farms in the fjords were due to high salinity, we also sampled fish from two indoor facilities (Farms 13 and 14) that use full‐salinity seawater. In addition, samples were collected from two land‐based freshwater facilities (Farms 10 and 15), where fish were kept in large outdoor tanks covered with bird netting. The results are shown in Figure 3.

Fish kept indoors in seawater (Farms 13 and 14) had similarly low concentrations of D3 and 25(OH)D3 as those reared indoors in freshwater (values from Farms 1 and 3, shown in Table 2). Similarly, fish kept in outdoor freshwater tanks covered only with bird netting (Farms 10 and 15) exhibited high concentrations of D3 and 25(OH)D3, comparable to those observed in outdoor net pens in full‐salinity seawater (values from Farms 2, 4, 5, 6, 7, 8, 9, 11, and 12, shown in Table 2), and significantly higher than the indoor farms. However, concentrations of 1,25(OH)_2_D3 were significantly lower at the outdoor farms.

### 3.4. Seasonal Effects

**Figure 4:**
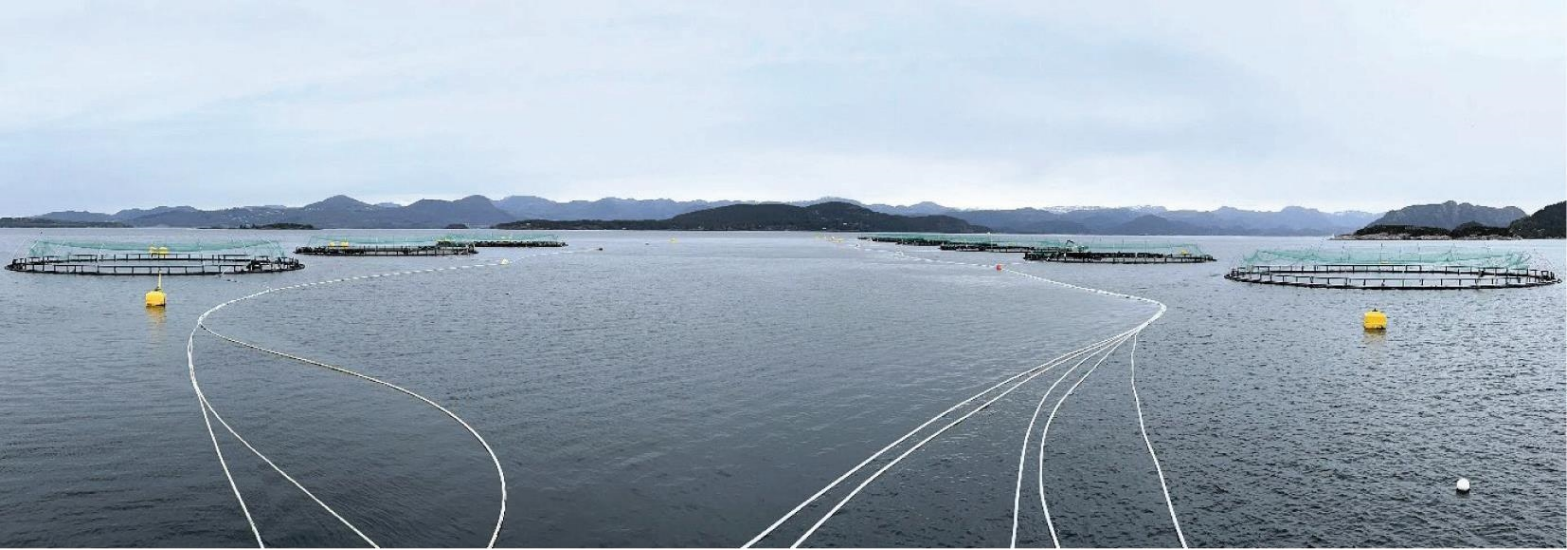
Store Teistholmen (Farm 7), Rogaland County, Southern Norway. The farm comprised eight net pens, each with a depth of 35 m and a circumference of 160 m. Each pen housed ~150,000 fish.

Based on the data collected so far, it was decided to monitor the effect of changes in daylight at a sea‐based facility with open net pens. Since Skretting holds an R&D license at Farm 7 (Store Teistholmen, Rogaland County, Southern Norway, Figure 4) and therefore has unrestricted access, this farm was chosen for the longitudinal study.

**Figure 5:**
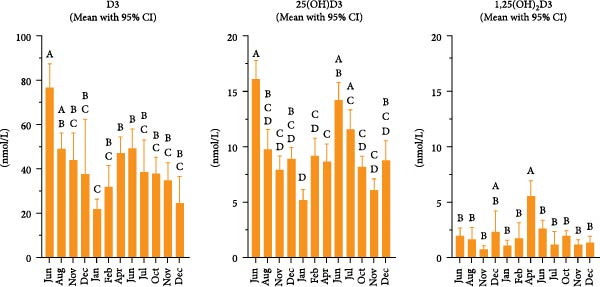
Vitamin D metabolites in plasma from fish reared outdoors in seawater in open pens (Farm 7, Store Teistholmen, Rogaland County, Southern Norway). The site was followed over a period of 18 months. The dataset is based on two fish groups. The first group was followed from June 2023 until harvest in February 2024. Another group was transferred in March 2024 and followed from April 2024 until December 2024. Plotted data are mean values with 95% confidence intervals. Mean values per sampling were compared using one‐way ANOVA and Tukey’s multiple comparisons test. Samplings with different letters indicate statistically significant differences in mean values (*p* < 0.05).

Twelve samplings were done between June 2023 and December 2024. The results can be seen in Figure 5. A clear seasonal effect was found for both D3 and 25(OH)D3. The level of these metabolites was about three times lower in January compared to the levels measured in June. Day length at Farm 7 was 6 h and 11 min at the winter solstice and 18 h and 29 min at the summer solstice.

### 3.5. Effect of Daylight on Whole‐Body Content of Vitamin D

As a follow‐up experiment, we aimed to investigate whether the observed differences in circulating vitamin D levels were also reflected in stored vitamin D within the body. During this period, we became aware of a land‐based hatchery (Farm 10) that was planning to divide a group of juvenile fish reared indoors, transferring half to outdoor tanks while keeping the remainder indoors. This setup provided a valuable opportunity for comparative analysis. The fish were initially kept indoors in freshwater and shielded from sunlight until they reached ~25 g in weight. On August 6th, the group was split, with half moved to a large outdoor freshwater tank exposed to daylight, while the other half remained indoors. Both groups were fed the same commercial diet before and after the split. After 52 days, on September 27th, five fish from each group were sampled.

**Figure 6:**
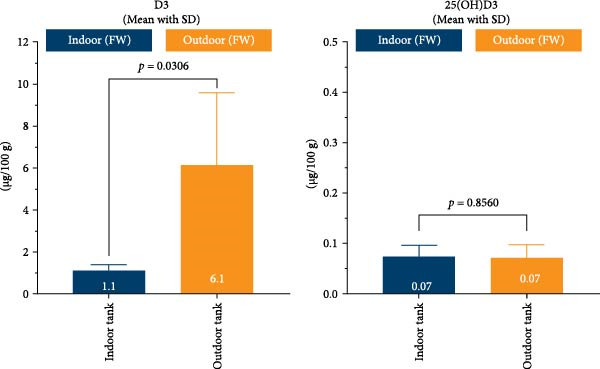
Vitamin D metabolites in whole‐body analyses from juvenile fish reared indoors in fresh water versus fish from the same fish group, fed the same commercial feed, but kept in an outdoor freshwater tank for 52 days. Mean with standard deviation. Indoor and outdoor groups were compared by Welch’s *t*‐test (*p*‐values plotted on graph).

The average weight of the five random fish from the outdoor tank was 70 g whereas the average weight of the five random fish from the indoor tank was 74 g. All 10 fish were homogenized, and the level of vitamin D metabolites was measured in the whole‐body homogenate from each individual fish. Results are shown in Figure 6. Fish exposed to daylight for 52 days had about 5 times higher levels of D3 in their whole bodies compared to fish reared indoors. The level of 25(OH)D3 did not differ. Vitamin D levels in tissue samples are expressed in µg/100 g to facilitate comparison with values reported in nutritional studies and food composition databases. For reference, a plasma concentration of 50 nmol/L of vitamin D3 corresponds to ~19.2 µg/L in plasma, assuming a plasma density of 1.0 g/mL.

## 4. Discussion

### 4.1. Level of Vitamin D Metabolites in Plasma

The initial screening of 12 fish farms revealed that plasma D3 levels in Atlantic salmon could reach as high as 300 nmol per liter, ~3–5 times higher than typical levels found in humans [9]. Interestingly, D3 levels were consistently higher than 25(OH)D3 levels, in contrast to humans, where 25(OH)D3 is the dominating vitamin D metabolite in plasma [9]. The level of 1,25(OH)_2_D3 ranged from 1 to 10 nmol per liter, also considerably higher than in humans but in line with earlier findings from Eide Graf et al. [26] and Lock et al. [27], who reported average ranges of 0.5–1.5 nmol per liter and 0.5–4.2 nmol per liter, respectively.

Lock et al. [27] reported that 1,25(OH)_2_D3 levels increased to 4.2 nmol per liter in seawater‐adapted juvenile Atlantic salmon when the fish were moved from freshwater to a 50:50 freshwater–seawater mixture. In our study, we observed elevated levels of 1,25(OH)_2_D3 in fish‐group 3 (Table 3), both before and after seawater transfer (also reported in Table 2 for Farm 11). However, no clear increase was observed around the time of transfer in groups 1 and 2 (Table 3), as these fish had already been in full salinity seawater within the indoor facility for several months before being moved to open sea pens. Fish at Farm 6, sampled approximately 4 weeks after sea transfer, also showed high levels of 1,25(OH)_2_D3. At Farm 7, which was monitored over 18 months, the highest level of 1,25(OH)_2_D3 was observed in April, approximately 4 weeks after the seawater transfer of the second fish group at this site (Figure 5). These findings align well with previous research suggesting that 1,25(OH)_2_D3 levels rise during the seawater adaptation phase in salmonids [27, 31].

The more than 20‐fold difference in circulating D3 between the lowest and highest fish groups was unexpected. As part of the annual governmental monitoring program, the Norwegian Institute of Marine Research has previously examined D3 levels in commercial fish feed in Norway. In an analysis of 85 feed samples, the average D3 content was found to be 0.13 mg per kg, with a minimum of 0.06 mg per kg and a maximum of 0.50 mg per kg [32]. Based on these data, a difference of more than eightfold in circulating D3 levels was not expected, and in practice, even smaller differences would be anticipated, given that most fish farms receive new feed batches several times per month. The surprisingly large variation could neither be explained by the timing of sampling after feeding, as significant variation was also observed in plasma 25(OH)D3 levels, a metabolite known for its long half‐life and relative insensitivity to postprandial effects [17].

A distinct pattern in vitamin D levels emerged when comparing indoor and outdoor farms. To confirm that the observed differences were not due to genetic variation among fish groups, three groups were monitored before and after being transferred to outdoor net pens in the sea (Table 3). These analyses confirmed that vitamin D metabolism changed significantly after the transfer. To assess whether increased salinity played a role, fish from Farm 10 and Farms 13–15 were sampled, allowing comparisons of vitamin D levels in fish reared outdoors in freshwater and indoors in seawater. Again, outdoor‐reared fish had high vitamin D levels, while indoor‐reared fish had low levels, demonstrating that the differences observed before and after transfer to open sea pens were unrelated to salinity changes (Figure 3).

One remaining plausible explanation was that the increased vitamin D levels resulted from sunlight exposure. If true, vitamin D levels could also be expected to be influenced by daylight hours. Farm 7, located in Southern Norway, was monitored over 18 months to investigate this hypothesis, and the results (Figure 5) confirmed a seasonal pattern: circulating vitamin D levels declined during fall and rose again in spring. During winter, levels dropped nearly to those observed in fish kept indoors. This seasonal effect is expected to be even more pronounced in Northern Norway.

To test whether sunlight not only increased circulating vitamin D metabolites but also increased stored vitamin D, juvenile fish were divided into two groups. One was moved to an outdoor tank covered only by bird netting, while the other remained indoors. Both groups were fed the same commercial feed during the 52‐day study. The outdoor group showed a fivefold increase in stored vitamin D levels, demonstrating that sunlight substantially enhances vitamin D storage in the body (Figure 6). These findings indicate that sunlight‐induced vitamin D synthesis is the primary source of vitamin D for farmed Atlantic salmon being fed commercial feed in outdoor facilities.

Until recently, the consensus in fish nutrition has been that fish do not synthesize vitamin D and must rely entirely on dietary sources to meet their requirements [23]. However, Pierens and Fraser [8] detected 7‐dehydrocholesterol in the skin of both rainbow trout and Atlantic salmon. When rainbow trout were exposed to simulated sunlight (290–1200 nm) or blue light (380–480 nm), with light passing through 5 cm of water at 12°C, D3 formation was detected in the skin. Fish were exposed to light in two 30‐min pulses, with simulated sunlight giving the strongest and fastest response. D3 level in the skin of the two fish groups rose to ~1.8 µg per 100 g and 0.5 µg per 100 g after the first pulse and further to ~2.5 µg per 100 g and 2.0 µg per 100 g, respectively, after the second pulse. In a subsequent experiment, isolated skin from both trout and rats was exposed to blue light (380–480 nm). Again, D3 formation was detected in trout skin, but none was found in rat skin, suggesting that blue light, which contains less energy than UVB light, was sufficient to convert 7‐dehydrocholesterol to D3 in trout skin but not in rat skin [8]. Interestingly, these findings indicate that wavelengths longer than classical UVB, including UVA and visible blue light, may also contribute to vitamin D synthesis in the skin of fish. The effect of UV light has also been investigated in Mozambique tilapia by Rao and Raghuramulu [33]. They exposed fish (200–250 g) kept in small freshwater aquariums of 15 cm depth to UV light (300 nm) for 15 h and were able to detect a significant increase in vitamin D in the whole body.

Despite these interesting findings in shallow aquariums using artificial UV light, it has not been widely accepted that fish can synthesize vitamin D when exposed to natural sunlight. This skepticism largely arises because the penetration of UV light in water is perceived to be very limited. Early studies reported that only 1% of surface UV radiation (300–400 nm) reached a depth of 25 m in the sea [34]. Another study, which investigated the penetration of solar radiation at various stations in the central subtropical Atlantic Ocean, found that UVB light (280–315 nm) reached depths of 12–31 m, depending on the optical properties of the water, while UVA light (315–400 nm) reached depths of 29–65 m [35]. More recently, Lee et al. [36] demonstrated that UV light might penetrate deeper than previously thought. They measured that ~10% of surface UVA light (360 nm) was still present at depths of 50–70 m in oligotrophic waters. Taken together with the findings of Pierens and Fraser [8], who observed the formation of vitamin D in the skin of trout exposed to artificial light with wavelengths of 380–480 nm, this helps explain how natural sunlight may contribute to endogenous vitamin D synthesis in fish even at water depths where UVB penetration is limited. This further suggests that freshwater species and epipelagic marine species inhabiting the sunlit uppermost layers of the ocean are exposed to sufficient UV light in their natural habitats, making the conversion of 7‐dehydrocholesterol to cholecalciferol in the skin feasible.

It has been suggested that vitamin D in fish may derive from UV‐induced synthesis in zooplankton and phytoplankton, which is then transferred through the aquatic food chain [23, 24]. To assess whether plankton in the fjords could explain the observed increase after transfer in the present study, we performed a simple mass‐balance calculation. Commercial feed typically contains ~5200 IU/kg vitamin D [32], and using an estimate of 1% feed intake during summer months for fish around 1 kg, daily feed intake in a pen with 200,000 fish would be ~2000 kg, providing 10.4 million IU/day. Plasma vitamin D levels increased six‐ to 11‐fold after transfer to outdoor pens (Table 3). Using the conservative lower end (sixfold) for the calculation, the daily vitamin D contribution from algae would need to be ~5 times higher than that from feed, or roughly 52 million IU/day. Reported vitamin D levels in plankton are sparse and vary widely. Ljubic et al. [37] performed laboratory experiments with four different strains of microalgae exposed to artificial UVB light in high doses. Of the four species, exclusively *Nannochloropsis oceanica* was able to produce vitamin D3 up to 1000 ng/g dry weight. Freshwater plankton from India has been measured at up to 800 ng/g dry weight in phytoplankton and 2700 ng/g dry weight in zooplankton; the latter is equivalent to 2.7 mg/kg or ~108 000 IU/kg dry weight [38]. These conditions are unlikely to reflect marine fjord conditions at Norwegian latitudes, where UV exposure is much lower. Field measurements in marine environments more relevant to our study found very low or undetectable vitamin D levels in zooplankton: Kenny et al. [39] reported no detectable vitamin D in zooplankton from active bowhead whale feeding sites in Alaska, and van der Meeren et al. [40] found only trace or undetectable levels in copepods near Austevoll, Norway. Nevertheless, even using the high‐end Indian numbers for illustration, salmon would need to consume about 481 kg of additional dry plankton per day to reach 52 million IU (calculated as 52,000, 000 IU ÷ 108,000 IU/kg). Assuming a dry matter content of 20%, this corresponds to ~2405 kg wet plankton per day, more than the fish’s normal daily feed intake of 2000 kg. Salmonids are not filter‐feeders, and it seems very unlikely that their intestinal tract could suddenly process such large additional volumes of planktonic biomass. These simplified mass‐balance considerations therefore strongly suggest that the increased vitamin D status observed in the present study cannot be explained by intake of plankton, even under highly optimistic assumptions. Taken together, our findings provide strong evidence that sunlight‐induced endogenous synthesis is responsible for the elevated vitamin D levels observed in outdoor‐farmed salmon. This provides fundamentally new insights into vitamin D metabolism in Atlantic salmon, highlighting the biological relevance of natural sunlight exposure for fish fed standard commercial diets.

The biological importance of the low circulating vitamin D levels observed in fish from indoor facilities and outdoor farms during winter needs further investigation. The increased levels of 1,25(OH)_2_D3 in the indoor farms (Farms 13 and 14, Table 2) may indicate vitamin D deficiency, as elevated concentrations of 1,25(OH)_2_D3 have been reported in vitamin D‐deficient humans^13^. However, this finding requires further investigation and may also be influenced by other physiological or environmental factors. It remains unclear whether the low levels of 25(OH)D3 and D3 could have adverse effects on the fish. However, the naturally high circulating levels found during summertime suggest potential benefits of elevated vitamin D status. In biological systems, sustained synthesis of a metabolically costly compound at high levels typically reflects evolutionary or physiological relevance, suggesting that elevated vitamin D status may provide a biological advantage.

Atlantic salmon is a migratory fish that spawns in rivers. After hatching, the juvenile salmon spend about 1–3 years in rivers and lakes before migrating to the ocean. Migration typically occurs in late spring, and given their route through shallow and sunlit rivers, juvenile fish can be expected to have high vitamin D status when reaching the sea. This contrasts with modern farming practices, where most juvenile salmon are kept indoors and have a low vitamin D status when transferred to the sea, as demonstrated in the current study (Table 3).

Vitamin D has been shown to play an important role in the tight junction proteins responsible for sealing the intercellular space between epithelial cells, and vitamin D deficiency has been linked to compromised barrier function in the intestine, lungs, and skin in humans [41–43]. An intact barrier function is crucial for fish to minimize dehydration when migrating from freshwater to seawater, and a healthy skin barrier is essential for protection against the vast amounts of pathogenic bacteria present in seawater, such as *Tenacibaculum* spp. Interestingly, it is well known that farmed salmon are particularly vulnerable to infections from *Tenacibaculum* during the first weeks after being transferred to seawater [44].

During winter, farmed Atlantic salmon are susceptible to winter ulcer bacteria such as *Moritella viscosa* and *Tenacibaculum* spp. [44]. However, skin health improves as daylight increases during spring, sometimes even before water temperatures begin to rise. This improvement may be due to factors other than increased vitamin D, but based on the current findings, it would be worthwhile exploring if higher vitamin D levels in the feed could benefit skin health during winter.

Conversely, the high levels of vitamin D observed during summertime suggest that additional supplementation during this period may not be necessary for fish reared in outdoor environments. A future experiment without extra supplementation of vitamin D in the feed of salmon kept in outdoor pens during summertime could also serve to further demonstrate the importance and magnitude of endogenous vitamin D production induced by natural sunlight.

Finally, the new findings show that salmon kept indoors until slaughter can be expected to have significantly lower levels of vitamin D in their fillets compared to those farmed in outdoor facilities. Addressing this issue, whether by using UV lamps or increasing vitamin D levels in the feed, is essential to ensure that salmon from indoor facilities provide consumers with the same vitamin D content as salmon from outdoor farms.

The discovery that fish can synthesize vitamin D through sunlight exposure could have wide‐ranging implications for farming practices and the nutritional quality of farmed fish.

It is important to acknowledge several limitations of the study. This research was conducted in collaboration with 15 fish farms, each operating under practical production conditions. While this provided a valuable opportunity to investigate vitamin D status across diverse environments, fish groups, and rearing practices, it also introduced certain constraints. Within the scope of this study, it was unfortunately not possible to obtain detailed information on feed composition (including vitamin D content), feed intake, or complete baseline vitamin D levels. These parameters are highly relevant for interpreting physiological responses and nutritional dynamics, and we recognize that their absence limits the depth of analysis.

For future research, we suggest conducting a controlled experiment with and without daylight exposure, where the vitamin D level in the feed is kept similar and well characterized, and where plankton availability is either quantified or minimized through appropriate water treatments. Using fish of comparable size and monitoring both plasma and whole‐body vitamin D concentrations over time would allow construction of detailed time curves, providing further insight into the kinetics of endogenous vitamin D synthesis and how rapidly circulating and stored levels are affected by sunlight.

Nevertheless, the present study provides novel and unexpected insights into vitamin D metabolism in Atlantic salmon under varying environmental conditions, and we believe these findings highlight important considerations for aquaculture practices and provide a strong foundation for future investigations.

## 5. Conclusion

This study presents novel evidence that Atlantic salmon can synthesize vitamin D endogenously when exposed to natural sunlight. Plasma analyses across 12 fish farms revealed unexpectedly high and variable levels of vitamin D metabolites, particularly D3, which in some cases exceeded human levels by a factor of 3–5. A clear pattern emerged linking higher vitamin D levels to outdoor rearing, independent of diet, genetics, or salinity. Seasonal variation in plasma D3 levels, with marked declines in winter and increases in spring, further supported a role for sunlight. A controlled trial confirmed that sunlight exposure significantly enhances vitamin D content in whole fish, reinforcing the conclusion that sunlight‐induced synthesis is the dominant source of vitamin D in farmed salmon reared outdoors on standard diets.

These findings challenge the long‐held belief that fish are entirely dependent on dietary vitamin D. Historical skepticism, based on the limited penetration of UV light in water, is contradicted by more recent measurements suggesting that sufficient UV light could be available in the upper water column to permit dermal synthesis. The implications are significant: salmon reared entirely indoors or during periods of low daylight may be at risk of developing a suboptimal vitamin D status, which could potentially compromise skin integrity and increase vulnerability to pathogens such as *Tenacibaculum* spp. Moreover, vitamin D levels in the flesh of indoor‐farmed salmon are likely to be substantially lower than in outdoor‐reared fish, raising concerns about nutritional quality for consumers. Strategies such as UV supplementation or dietary fortification should be considered to address these concerns when fish are exposed to limited sunlight.

## Ethics Statement

All handling of fish complied with the Guidelines of EU legislation (Directive 2010/63/EU) and Norwegian legislation (LOV‐2009‐06‐19‐97).

## Disclosure

All authors read and approved the final version of the manuscript.

## Conflicts of Interest

Skretting is a fish feed company, Nordlaks is a fish farming company, and FishLab offers vitamin D analyses in fish. The authors declare no conflicts of interest .

## Author Contributions

C.A.H., J.R., E.W., and D.L.K. conceived the study and designed the sampling plan. C.A.H., K.B., F.K‐G., E.H., J.H., M.S., K.M.B., L.L., T.M., I.S., and H.B. collected all field samples. T. B., C.B., and D.L.K. developed analytical methods for sample preparation and LC‐MS/MS and performed the analysis. C.A.H., J.M., C.B., and D.L.K. interpreted the results. C.B. and D.L.K. wrote the manuscript, with input from C.A.H., F.K.‐G., E.H., K.M.B., L.L., T.M., I.S., J.M., and H.B.

## Funding

This study was partly funded by the Research Council of Norway (Grant No. 328674).

## Supporting Information

Additional supporting information can be found online in the Supporting Information section.

## Supporting information


**Supporting Information** Table S1: Level of vitamin D metabolites in plasma (nmol per liter) from Atlantic salmon sampled at 12 different fish farms. Table S2: Level of vitamin D metabolites in plasma (nmol per liter) in three different fish groups sampled before and after transfer to outdoor pens. Table S3: Vitamin D metabolites in plasma from fish reared indoors in seawater (SW) and outdoors in freshwater (FW), reported in nmol per liter. Table S4: Vitamin D metabolites in plasma from fish reared outdoors in seawater in open pens (Farm 7, Store Teistholmen, Rogaland County, Southern Norway). Table S5: Vitamin D metabolites in whole‐body analyses from juvenile fish reared indoors in fresh water versus fish from the same fish group, fed the same commercial feed, but kept in an outdoor freshwater tank for 52 days.

## Data Availability

Additional supporting information can be found online in the supporting data file.

## References

[1] McCollum E. V. , Simmonds N. , Becker J. E. , and Shipley P. G. , Studies on Experimental Rickets: XXI. An Experimental Demonstration of the Existence of a Vitamin Which Promotes Calcium Deposition, Journal of Biological Chemistry. (1922) 53, no. 2, 293–312, 10.1016/S0021-9258(18)85783-0. 11991957

[2] Jones G. , 100 Years of Vitamin D: Historical Aspects of Vitamin D, Endocrine Connections. (2022) 11, no. 4, 10.1530/EC-21-0594, e210594. 35245207 PMC9066576

[3] Windaus A. , Schenck F. , and Werder F. T. , Über das Antirachitisch Wirksame Bestrahlungsprodukt ans 7-Dehydro-Cholesterin, Hoppe-Seyler’s Zeitschrift für Physiologische Chemie. (1936) 241, no. 1–3, 100–103, 10.1515/bchm2.1936.241.1-3.100, 2-s2.0-84941394487.

[4] Zerwekh J. E. , Blood Biomarkers of Vitamin D Status, The American Journal of Clinical Nutrition. (2008) 87, no. 4, 1087S–1091S, 10.1093/ajcn/87.4.1087S. 18400739

[5] DeLuca H. F. , Evolution of Our Understanding of Vitamin D, Nutrition Reviews. (2008) 66, S73–S87, 10.1111/j.1753-4887.2008.00105.x, 2-s2.0-52649174229. 18844850

[6] Cutolo M. , Smith V. , Paolino S. , and Gotelli E. , Involvement of the Secosteroid Vitamin D in Autoimmune Rheumatic Diseases and COVID-19, Nature Reviews Rheumatology. (2023) 19, no. 5, 265–287, 10.1038/s41584-023-00944-2. 36977791 PMC10043872

[7] Holick M. F. , Chen T. C. , Lu Z. , and Sauter E. , Vitamin D and Skin Physiology: A D-Lightful Story, Journal of Bone and Mineral Research. (2007) 22, no. S2, V28–V33, 10.1359/jbmr.07s211, 2-s2.0-39749113142. 18290718

[8] Pierens S. L. and Fraser D. R. , The Origin and Metabolism of Vitamin D in Rainbow Trout, The Journal of Steroid Biochemistry and Molecular Biology. (2015) 145, 58–64, 10.1016/j.jsbmb.2014.10.005, 2-s2.0-84908173372. 25305412

[9] Hollis B. W. , Wagner C. L. , Drezner M. K. , and Binkley N. C. , Circulating Vitamin D3 and 25-Hydroxyvitamin D in Humans: An Important Tool to Define Adequate Nutritional Vitamin D Status, The Journal of Steroid Biochemistry and Molecular Biology. (2007) 103, no. 3–5, 631–634, 10.1016/j.jsbmb.2006.12.066, 2-s2.0-33947100939. 17218096 PMC1868557

[10] Hollis B. W. and Wagner C. L. , The Role of the Parent Compound Vitamin D With Respect to Metabolism and Function: Why Clinical Dose Intervals can Affect Clinical Outcomes, The Journal of Clinical Endocrinology & Metabolism. (2013) 98, no. 12, 4619–4628, 10.1210/jc.2013-2653, 2-s2.0-84889867812. 24106283 PMC3849670

[11] Jones G. , Pharmacokinetics of Vitamin D Toxicity, The American Journal of Clinical Nutrition. (2008) 88, no. 2, 582S–586S, 10.1093/ajcn/88.2.582S. 18689406

[12] Xu S. , Ni R. , and Lv L. , et al.Simultaneous Determination of Vitamin D Metabolites 25(OH)D3 and 1*α*,25(OH)2D3 in Human Plasma Using Liquid Chromatography Tandem Mass Spectrometry, Journal of Mass Spectrometry and Advances in the Clinical Lab. (2022) 24, 65–79, 10.1016/j.jmsacl.2022.04.001. 35572785 PMC9093011

[13] Holick M. F. , Vitamin D Status: Measurement, Interpretation, and Clinical Application, Annals of Epidemiology. (2009) 19, no. 2, 73–78, 10.1016/j.annepidem.2007.12.001, 2-s2.0-58949093000. 18329892 PMC2665033

[14] Wagner C. L. , Taylor S. N. , and Hollis B. W. , Does Vitamin D Make the World Go “Round”?, Breastfeeding Medicine: The Official Journal of the Academy of Breastfeeding Medicine. (2008) 3, no. 4, 239–250, 10.1089/bfm.2008.9984, 2-s2.0-57749105243. 19086827 PMC2981372

[15] Meems L. M. G. , Brouwers F. P. , and Joosten M. M. , et al.Plasma Calcidiol, Calcitriol, and Parathyroid Hormone and Risk of New Onset Heart Failure in a Population-Based Cohort Study, ESC Heart Failure. (2016) 3, no. 3, 189–197, 10.1002/ehf2.12089, 2-s2.0-85032809921. 27818783 PMC5074250

[16] Mezquita-Raya P. , Muñoz-Torres M. , and De Dios Luna J. , et al.Bone Density, and Bone Metabolism in Healthy Postmenopausal Women, Journal of Bone and Mineral Research. (2001) 16, no. 8, 1408–1415, 10.1359/jbmr.2001.16.8.1408, 2-s2.0-0034919391. 11499863

[17] McCourt A. F. , Mulrooney S. L. , O’Neill G. J. , O’Riordan E. D. , and O’Sullivan A. M. , Postprandial 25-Hydroxyvitamin D Response Varies According to the Lipid Composition of a Vitamin D3 Fortified Dairy Drink, International Journal of Food Sciences and Nutrition. (2022) 73, no. 3, 396–406, 10.1080/09637486.2021.1984400. 34615419

[18] Holick M. F. , Resurrection of Vitamin D Deficiency and Rickets, The Journal of Clinical Investigation. (2006) 116, no. 8, 2062–2072, 10.1172/JCI29449, 2-s2.0-33746741731. 16886050 PMC1523417

[19] Vieth R. , Vitamin D Supplementation, 25-Hydroxyvitamin D Concentrations, and Safety ^∗^ , The American Journal of Clinical Nutrition. (1999) 69, no. 5, 842–856, 10.1093/ajcn/69.5.842. 10232622

[20] DeLuca H. F. , Overview of General Physiologic Features and Functions of Vitamin D, The American Journal of Clinical Nutrition. (2004) 80, no. 6, 1689S–1696S, 10.1093/ajcn/80.6.1689S. 15585789

[21] Jones G. , Strugnell S. A. , and DeLuca H. F. , Current Understanding of the Molecular Actions of Vitamin D, Physiological Reviews. (1998) 78, no. 4, 1193–1231, 10.1152/physrev.1998.78.4.1193, 2-s2.0-0031755168. 9790574

[22] Gil Á. , Plaza-Diaz J. , and Mesa M. D. , Vitamin D: Classic and Novel Actions, Annals of Nutrition and Metabolism. (2018) 72, no. 2, 87–95, 10.1159/000486536, 2-s2.0-85040744923. 29346788

[23] Lock E.-J. , Waagbø R. , Wendelaar Bonga S. , and Flik G. , The Significance of Vitamin D for Fish: A Review, Aquaculture Nutrition. (2010) 16, no. 1, 100–116, 10.1111/j.1365-2095.2009.00722.x, 2-s2.0-74249093398.

[24] Fraser D. R. , Chapter 2-Evolutionary Biology: Mysteries of Vitamin D in Fish, Vitamin D, 2018, 4th edition., Academic Press, 13–27, 10.1016/B978-0-12-809965-0.00002-1, 2-s2.0-85042551983.

[25] Moxness Reksten A. , Ho Q. T. , and Nøstbakken O. J. , et al.Temporal Variations in the Nutrient Content of Norwegian Farmed Atlantic Salmon (*Salmo salar*), 2005-2020, Food Chemistry. (2022) 373, 10.1016/j.foodchem.2021.131445, 131445. 34731805

[26] Eide Graff I. , Stefansson S. O. , Aksnes L. , and Lie Ø. , Plasma Levels of Vitamin D3 Metabolites During Parr-Smolt Transformation of Atlantic Salmon, Aquaculture. (2004) 240, no. 1, 617–622, 10.1016/j.aquaculture.2004.06.025, 2-s2.0-4744359363.

[27] Lock E. J. , Ornsrud R. , Aksnes L. , Spanings F.a T. , Waagbø R. , and Flik G. , The Vitamin D Receptor and Its Ligand 1alpha,25-Dihydroxyvitamin D3 in Atlantic Salmon (*Salmo salar*), The Journal of Endocrinology. (2007) 193, no. 3, 459–471, 10.1677/JOE-06-0198, 2-s2.0-34447100776. 17535883

[28] Ruxton G. D. , The Unequal Variance t-Test Is an Underused Alternative to Student’s t-Test and the Mann–Whitney U Test, Behavioral Ecology. (2006) 17, no. 4, 688–690, 10.1093/beheco/ark016, 2-s2.0-33745595610.

[29] Delacre M. , Lakens D. , and Leys C. , Why Psychologists Should by Default Use Welch’s t-Test Instead of Student’s t-Test, International Review of Social Psychology. (2017) 30, no. 1, 92–101, 10.5334/irsp.82, 2-s2.0-85019141079.

[30] Keitel-Gröner F. , Hoel E. , and Husebø C. , et al.Haematological and Biochemical Reference Intervals Towards a Proactive Health Monitoring Approach in Norwegian Atlantic Salmon Farming, Journal of Fish Diseases. (2025) 48, no. 9, 10.1111/jfd.14036. PMC12368258 39445767

[31] Larsson D. , Nemere I. , Aksnes L. , and Sundell K. , Environmental Salinity Regulates Receptor Expression, Cellular Effects, and Circulating Levels of Two Antagonizing Hormones, 1,25-Dihydroxyvitamin D3 and 24,25-Dihydroxyvitamin D3 Rainbow Trout, Endocrinology. (2003) 144, no. 2, 559–566, 10.1210/en.2002-220779, 2-s2.0-0037320322. 12538617

[32] Sele V. , Berntssen M. , Philip A. , Lundebye A.-K. , Lie K. K. , and Espe M. , Monitoring Program for Fish Feed, Norwegian institute of Marine Research, Annual report for samples retrieved in 2020.

[33] Rao D. S. and Raghuramulu N. , Vitamin D3 in Tilapia Mossambica: Relevance of Photochemical Synthesis, Journal of Nutritional Science and Vitaminology. (1997) 43, no. 4, 425–433, 10.3177/jnsv.43.425. 9328861

[34] Fleischmann E. M. , The Measurement and Penetration of Ultraviolet Radiation Into Tropical Marine Water, Limnology and Oceanography. (1989) 34, no. 8, 1623–1629, 10.4319/lo.1989.34.8.1623, 2-s2.0-84986798856.

[35] Piazena H. , Perez-Rodrigues E. , Häder D.-P. , and Lopez-Figueroa F. , Penetration of Solar Radiation Into the Water Column of the Central Subtropical Atlantic Ocean—Optical Properties and Possible Biological Consequences, Deep Sea Research Part II: Topical Studies in Oceanography. (2002) 49, no. 17, 3513–3528, 10.1016/S0967-0645(02)00093-0, 2-s2.0-0036390475.

[36] Lee Z. , Hu C. , and Shang S. , et al.Penetration of UV-Visible Solar Radiation in the Global Oceans: Insights From Ocean Color Remote Sensing, Journal of Geophysical Research: Oceans. (2013) 118, no. 9, 4241–4255, 10.1002/jgrc.20308, 2-s2.0-84886271050.

[37] Ljubic A. , Jacobsen C. , Holdt S. L. , and Jakobsen J. , Microalgae *Nannochloropsis oceanica* as a Future New Natural Source of Vitamin D3, Food Chemistry. (2020) 320, 10.1016/j.foodchem.2020.126627, 126627. 32213421

[38] Sunita Rao D. and Raghuramulu N. , Food Chain as Origin of Vitamin D in Fish, Comparative Biochemistry and Physiology Part A: Physiology. (1996) 114, no. 1, 15–19, 10.1016/0300-9629(95)02024-1, 2-s2.0-0029890142.

[39] Kenny D. E. , O’Hara T. M. , Chen T. C. , Lu Z. , Tian X. , and Holick M. F. , Vitamin D Content in Alaskan Arctic Zooplankton, Fishes, and Marine Mammals, Zoo Biology. (2004) 23, no. 1, 33–43, 10.1002/zoo.10104, 2-s2.0-16544386098.

[40] van der Meeren T. , Olsen R. E. , Hamre K. , and Fyhn H. J. , Biochemical Composition of Copepods for Evaluation of Feed Quality in Production of Juvenile Marine Fish, Aquaculture. (2008) 274, no. 2–4, 375–397, 10.1016/j.aquaculture.2007.11.041, 2-s2.0-39049131106.

[41] Sun J. and Zhang Y.-G. , Vitamin D Receptor Influences Intestinal Barriers in Health and Disease, Cells. (2022) 11, no. 7, 10.3390/cells11071129, 1129. 35406694 PMC8997406

[42] Chen H. , Lu R. , Zhang Y. , and Sun J. , Vitamin D Receptor Deletion Leads to the Destruction of Tight and Adherens Junctions in Lungs, Tissue Barriers. (2018) 6, no. 4, 1–13, 10.1080/21688370.2018.1540904, 2-s2.0-85057297923. PMC6389123 30409076

[43] Trujillo-Paez J. V. , Peng G. , and Le Thanh Nguyen H. , et al.Calcitriol Modulates Epidermal Tight Junction Barrier Function in Human Keratinocytes, Journal of Dermatological Science. (2024) 114, no. 1, 13–23, 10.1016/j.jdermsci.2024.02.001. 38448341

[44] Spilsberg B. , Nilsen H. K. , and Tavornpanich S. , et al.Tenacibaculosis in Norwegian Atlantic Salmon (*Salmo salar*) Cage-Farmed in Cold Sea Water Is Primarily Associated With Tenacibaculum Finnmarkense Genomovar Finnmarkense, Journal of Fish Diseases. (2022) 45, no. 4, 523–534, 10.1111/jfd.13577. 35001372 PMC9303539

